# Leveraging Large Language Models to Generate Multiple-Choice Questions for Ophthalmology Education

**DOI:** 10.1001/jamaophthalmol.2025.3622

**Published:** 2025-10-16

**Authors:** Shahrzad Gholami, Daniel B. Mummert, Beth Wilson, Sarah Page, Rahul Dodhia, Juan M. Lavista Ferres, William B. Weeks, Dale E. Fajardo, Karine D. Bojikian

**Affiliations:** 1 AI for Good Research Lab, Microsoft, Redmond, Washington; 2 American Academy of Ophthalmology, San Francisco, California; 3 Department of Ophthalmology, University of Washington, Seattle

## Abstract

**Importance:**

Multiple choice questions (MCQs) are an important and integral component of ophthalmology residency training evaluation and board certification; however, high-quality questions are difficult and time-consuming to draft.

**Objective:**

To evaluate whether general-domain large language models (LLMs), particularly OpenAI’s Generative Pre-trained Transformer 4 (GPT-4), can reliably generate high-quality, novel, and readable MCQs comparable to those of a committee of experienced examination writers.

**Design, Setting, and Participants:**

This survey study, conducted from September 2024 to April 2025, assesses LLM performance in generating MCQs based on the American Academy of Ophthalmology (AAO) *Basic and Clinical Science Course* (*BCSC*) compared with a committee of human experts. Ten expert ophthalmologists, who were masked to the generation source, independently evaluated MCQs using a 10-point Likert scale (1 = extremely poor; 10 = criterion standard quality) across 5 criteria: appropriateness, clarity and specificity, relevance, discriminative power, and suitability for trainees.

**Intervention:**

Relevant *BCSC* content and AAO question-writing guidelines were input into GPT-4o via Microsoft’s Azure OpenAI Service, and structured prompts were used to generate MCQs.

**Main Outcomes and Measures:**

The primary outcomes were median scores and statistical comparisons using the bootstrapping method; string similarity scores based on Levenshtein distance (0-100, with 100 indicating identical content) between LLM-MCQs and the entire *BCSC* question bank; Flesch Reading Ease metric for readability; and intraclass correlation coefficient (ICC) for inter-rater agreement are reported.

**Results:**

The 10 graders had between 1 and 28 years of clinical experience in ophthalmology (median [IQR] experience, 6 years [3-15 years]). Questions generated by GPT-4 and a committee of experts received median scores of 9 and 9 in combined scores, appropriateness, clarity and specificity, and relevance (difference, 0; 95% CI, 0-0; *P* > .99); 8 and 9 in discriminative power (difference, 1; 95% CI, −1 to 1; *P* = .52); and 8 and 8 in suitability for trainees (difference, 0; 95% CI, −1 to 0; *P* > .99), respectively. Nearly 95% of LLM-MCQs had similarity scores less than 60, indicating most LLM-MCQs had limited or no resemblance to existing content. Interrater reliability was moderate (ICC, 0.63; *P* < .001), and mean (SD) readability scores were similar across sources (37.14 [22.54] vs 42.60 [22.84]; *P* > .99).

**Conclusions and Relevance:**

In this survey study, results indicate that an LLM could be used to develop ophthalmology board–style MCQs and expand examination banks to further support ophthalmology residency training. Despite most questions having a low similarity score, the quality, novelty, and readability of the LLM-generated questions need to be further assessed.

## Key Points

**Question:**

Can a general-domain large language model (LLM) generate high-quality ophthalmology multiple choice questions (MCQs)?

**Findings:**

In this survey study, as evaluated by 10 independent ophthalmologists, LLM-MCQs were comparable in quality to those authored by a committee of human experts across 5 key domains: appropriateness, clarity and specificity, relevance, discriminative power, and suitability for trainees. Additionally, nearly 95% of LLM-MCQs had a similarity score less than 60 (on a scale where 100 indicates identical content), suggesting that most LLM-MCQs had limited or no resemblance to existing content.

**Meaning:**

LLMs have the potential to enhance ophthalmology resident education through high-quality examination content generation.

## Teaser

This survey study evaluates whether general-domain large language models can reliably generate high-quality, novel, and readable multiple-choice questions that are comparable to those of a committee of experienced examination writers.

## Introduction

Advances in artificial intelligence (AI) and deep learning, particularly in ophthalmic imaging analyses, have generated significant success and enthusiasm within the ophthalmology community.^1,2,3^ AI has also shown considerable potential in assisting with disease diagnosis by analyzing a wide range of patient data, including medical imaging, laboratory results, and other clinical information.^2,3,4,5,6,7^ However, the full potential of AI in clinical care, decision-making, and residents’ training remains largely unexplored across all areas of medicine, including ophthalmology.^8,9,10^

OpenAI’s Generative Pre-trained Transformer 4 (GPT-4) is a general large language model (LLM) that processes extensive text data, learns complex language patterns, and responds naturally.^8,9,10^ Studies have shown that other GPT model variants, such as ChatGPT, perform well in clinical reasoning tasks, including the United States Medical Licensing Examination.^11^

Creating high-quality multiple-choice questions (MCQs) for ophthalmology training is a time-consuming process that demands significant pedagogical expertise and domain-specific precision. Expert clinicians must carefully craft each question to ensure clinical accuracy, educational relevance, and appropriate difficulty. This process requires not only deep knowledge of ophthalmology, but also an understanding of how to assess cognitive skills effectively, making it a challenging and resource-intensive task.

In this study, we evaluated the feasibility of using LLMs, specifically GPT-4, to generate board-style MCQs by conducting a masked survey with expert ophthalmologists to compare the quality of LLM-generated MCQs with those created by a committee of human experts from the American Academy of Ophthalmology (AAO).

## Methods

### Using LLMs to Generate Questions

In collaboration with the AAO and with their explicit permission to use copyrighted material, we used all 13 volumes of the *Basic and Clinical Science Course* (*BCSC*) textbook series. The content was securely processed through Microsoft’s Azure OpenAI Service, a US Health Insurance Portability and Accountability Act–compliant platform that facilitates the use of OpenAI’s models via protected application programming interfaces. This approach ensured that all data handling adhered to rigorous standards for privacy, security, and intellectual property protection. This study was conducted between September 2024 and April 2025.

**Figure 1.**
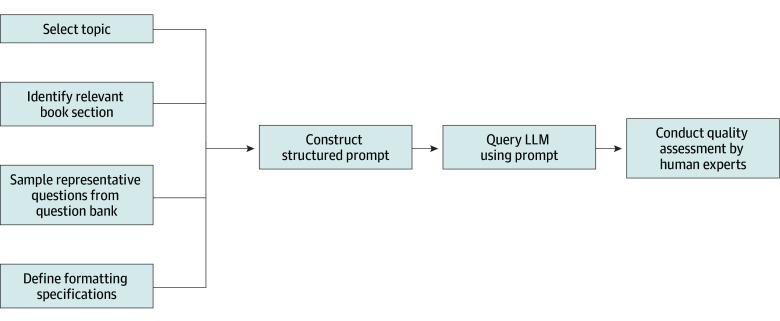
Workflow for Large Language Model (LLM)–Based Question Generation This flowchart illustrates the process for generating ophthalmology multiple-choice questions using an LLM. The workflow includes topic selection, identification of relevant content, sampling of representative questions, definition of formatting specifications, prompt construction, LLM querying, and postgeneration quality assessment by human experts.

To generate MCQs using an LLM, we used GPT-4o, the 2024-05-01-preview version with default parameters, and developed a structured prompting framework that incorporated key contextual elements, as illustrated in Figure 1. Each prompt included the topic name, relevant excerpts from the *BCSC*, and example questions drawn randomly from the AAO’s *BCSC* self-assessment program. Depending on availability, between 1 and 5 representative questions from the question bank were included in each prompt. The proprietary question-writing guidelines developed by the AAO were used to guide the model. In short, those guidelines generally align with recommendations from the National Board of Medical Examiners, where MCQs are written to have a question-based stem and 4 distractors. The items are reviewed and edited numerous times for consistency, clarity, and clinical accuracy by trained subject matter experts and staff based on these guidelines. An illustrative prompt is as follows: “Create 10 MCQs for the ophthalmology board exam based on this topic: <Topic> and this relevant textbook material: <Book Section>. Use this formatting: <Guideline>. Here are some example questions: <Example Questions from Question Bank>.”

Using this approach, we created a set of MCQs covering all 11 ophthalmology subspecialties. Considering the time needed to evaluate each question and the survey participants’ overall availability, we limited our final question set to 121 questions, nearly evenly split between those generated by LLMs (n = 64) and those written by a committee of human experts (n = 57). We randomly sampled questions to maintain a uniform subspecialty distribution across both LLM- and human-written content as much as the sample size allowed. Despite these efforts, minor differences in subspecialty representation remained. The proportion of questions per subspecialty was as follows (LLM vs human): pediatrics (9% vs 10%), refractives (9% vs 10%), retina (9% vs 10%), glaucoma (6% vs 10%), neuro-ophthalmology (9% vs 10%), pathology/oncology (11% vs 10%), cataract (6% vs 6%), uveitis (9% vs 10%), oculoplastics (9% vs 10%), cornea (6% vs 8%), and optics (14% vs 10%).

The human-written questions were drawn from the AAO’s *BCSC* Self-Assessment Program. These questions were written by trained ophthalmologists, reviewed by several experts, and adhere to best practices for high-stakes examinations.

### Survey to Compare Human Expert Committee– vs LLM-Generated Questions

We invited 24 ophthalmologists, including 13 of the Ophthalmic Knowledge Assessment Program (OKAP) examination and 11 of the Resident Self-Assessment Committee, to participate in our survey via the Alchemer survey platform. Participants were masked to the source of questions and invited to assess up to 121 MCQs using a 10-point Likert scale (where 1 is defined as extremely poor and 10 means that it is at the quality of a criterion-standard question) based on the following 5 key criteria: (1) appropriateness (technical and grammatical correctness, structure, and overall construction); (2) clarity and specificity (absence of ambiguity and adequacy of information); (3) relevance (alignment with clinical practice and context); (4) discriminative power (ability of the correct answer to stand out from plausible distractors); and (5) suitability for trainees (alignment with the Bloom taxonomy and ability to assess higher-order cognitive skills).

To evaluate differences between the 2 question-generation sources, we reported median scores for each evaluation criterion, aggregated median scores across all criteria, and median scores stratified by subspecialty. Following the American Association for Public Opinion Research guidelines, to estimate the difference in median scores between groups and assess statistical significance, we used a bootstrap approach with 10 000 resamples. In each iteration, samples were drawn with replacement from both groups, and the difference in medians was calculated. A 2-sided 95% confidence interval for the median difference was constructed using the percentile method. Additionally, a 2-sided *P* value was computed as the proportion of bootstrap iterations in which the absolute median difference was equal to or greater than the observed difference, testing the null hypothesis that there is no true difference in medians between groups. *P* < .05 was considered significant.

Participants were given 14 days to complete the survey and could save their progress and return later. All responses were anonymized, and participation was voluntary, with the option to discontinue at any time. The study was reviewed by Solutions IRB (protocol #0682) and verified as exempt from institutional review board review.

### Statistical Analysis

#### Compare the Similarity of LLM-Generated Questions With the *BCSC* Self-Assessment Question Bank

To compare LLM-generated questions with the entire question bank, we used the FuzzyWuzzy library, a Python library commonly used for fuzzy string matching, which involves identifying approximate rather than exact matches between text strings. It calculates a similarity score based on the Levenshtein distance—the minimum number of single-character edits (insertions, deletions, or substitutions) required to change one string into another.^12^ The library returns a similarity score ranging from 0 to 100, where 100 indicates an exact match and 0 represents no similarity. These similarity scores can be interpreted across different intervals: scores greater than 90 typically suggest near-identical strings, scores between 70 and 89 indicate a strong but not exact match, scores between 69 and 50 suggest moderate similarity, and scores less than 50 generally reflect low or negligible resemblance.

#### Assess the Readability of LLM-Generated Questions

To assess the readability of the questions, we applied the Flesch Reading Ease metric using the textstat library in Python (Python Software Foundation). This index assigns higher scores to texts that are easier to read, with values typically ranging from 0 to 100. Scores between 30 and 50 are generally appropriate for college-level readers. Scores less than 30 indicate very difficult text, best understood by individuals with advanced education.^13^ Statistical significance was assessed using the nonparametric Mann-Whitney *U* test to account for potential non-normality in the score distributions.

#### Analyze Interrater Agreement

To assess the consistency of expert evaluations, we computed Krippendorff α coefficients for each evaluation domain across both human- and LLM-generated questions (Table). Each question was independently rated by 10 expert graders using a 10-point Likert scale. Given the ordinal nature of this scale, Krippendorff α was calculated under the assumption of ordinal-level measurement, which accounts for the degree of disagreement between raters rather than treating all disagreements equally. This method is well-suited for multirater ordinal data and provides a robust estimate of interrater reliability for each question set. α Values range from −1 (perfect disagreement) to 1 (perfect agreement), with values near 0 indicating the level of agreement expected by chance. All calculations were performed using the Krippendorff Python package.^14^

**Table.** Krippendorff α Coefficients to Assess Interrater Agreement Across 5 Evaluation Domains for Human- and Machine-Generated Questions as Separate Sets, Based on Ratings From 10 Expert Graders

Domain	Human-written questions^a^	Machine-generated questions^a^
Appropriateness	0.016	−0.009
Clarity and specificity	−0.011	0.006
Suitability for trainees	0.120	0.077
Discriminative power	0.068	0.126
Relevance	0.059	0.094
Combined	0.072	0.088

^a^ Values closer to 1 indicate stronger agreement; negative values suggest disagreement beyond chance.

Overall interrater reliability was also assessed using the intraclass correlation coefficient (ICC). This approach was used to evaluate the consistency of ratings across multiple fixed raters who scored the same set of items including both human- and LLM-generated questions.^15^ Analyses were conducted using the pingouin Python package.

## Results

Ten ophthalmologists completed our survey, and their answers were included in the analyses. The graders had between 1 and 28 years of clinical experience in ophthalmology (median [IQR] experience, 6 years [3-15 years]), offering a broad yet experienced perspective for evaluation. The graders’ ophthalmology subspecialties included cataract/anterior segment, comprehensive ophthalmology, cornea/external disease, glaucoma, neuro-ophthalmology, retina, and uveitis.

**Figure 2.**
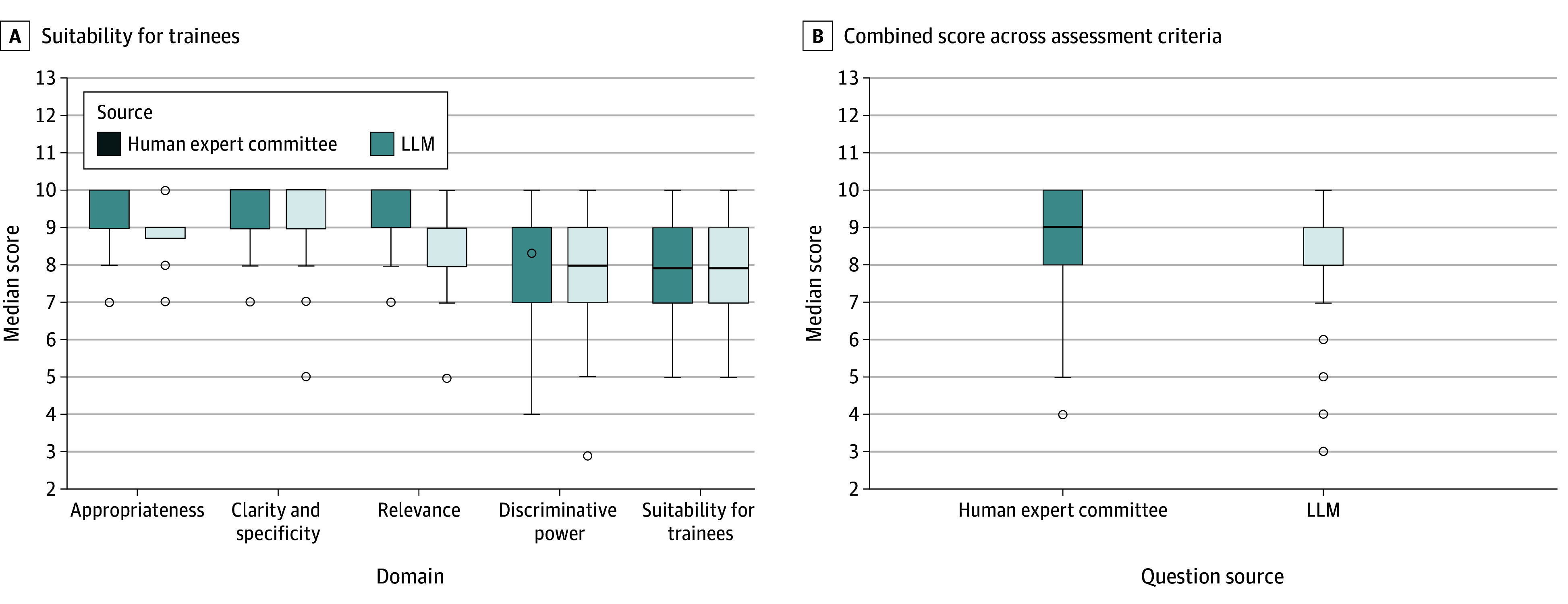
Distribution of Expert Median Scores Distribution of median scores assigned by 10 expert ophthalmologists who participated in the survey to compare question-generation methods based on the following criteria: appropriateness, clarity and specificity, relevance, discriminative power, and suitability for trainees (A), as well as the combined score across all assessment criteria (B). LLM indicates large language model.

The LLM-generated and human-written questions (examples in eTables 1 and 2 in Supplement 1) received comparable median scores across all evaluated domains. For appropriateness, both sets received a median score of 9 (difference, 0; 95% CI, 0-0; *P* > .99); for clarity and specificity, 9 vs 9 (difference, 0; 95% CI, 0-0; *P* > .99); for relevance, 9 vs 9 (difference, 0; 95% CI, 0-0; *P* > .99); for discriminative power, 8 vs 9 (difference, 1; 95% CI, –1 to 1; *P* = .52); and for suitability for trainees, 8 vs 8 (difference, 0; 95% CI, –1 to 0; *P* > .99). The combined median score assigned by the human expert committee to GPT-4– and human-written questions was also identical (9 vs 9; difference, 0; 95% CI, 0-0; *P* = .10). Figure 2 presents a comparison of the quality of human experts’ questions and those produced by the LLM. Box plots illustrate the distribution of scores for each generation method, providing insights into the variability and central tendency of the ratings.

**Figure 3.**
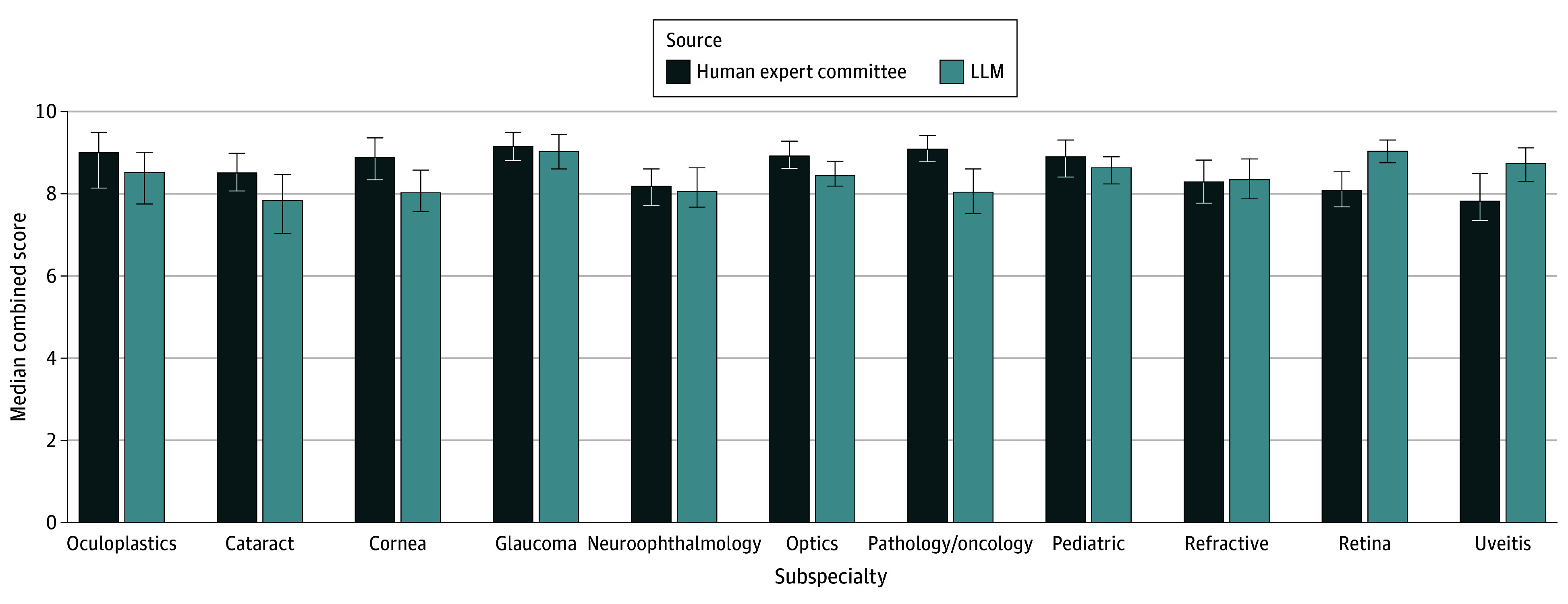
Combined Median Scores Across All Assessment Criteria Combined median scores across all assessment criteria for various subspecialties, as assigned by 10 expert ophthalmologists who participated in the survey comparing question-generation methods. LLM indicates large language model.

Figure 3 shows subspecialty-level comparisons between human- and machine-generated questions, which did not identify definitive differences in median scores. Retina (difference, 1.0; 95% CI, 0-1.0; *P* = .76) and uveitis (difference, 1.0; 95% CI, 0-2.0; *P* = .92) showed no definitively better outcomes for machine-generated content. Furthermore, no definitive differences were observed for machine-generated questions in oculoplastics (difference, −1.0; 95% CI, −1.5 to 0; *P* = .58), neuro-ophthalmology (difference, −1.0; 95% CI, −1.5 to 1.0; *P* = .44), cataract (difference, −1.0; 95% CI, −1.0 to 0; *P* = .58), or cornea (difference, −0.5; 95% CI, −2.0 to 0; *P* = .59). Several domains—including pathology/oncology, optics, glaucoma, and pediatric—demonstrated no difference in performance (all with difference of 0; 95% CI including 0; *P* > .99). In addition, refractive showed no definitive advantage for machine-generated items (difference, 0.5; 95% CI, −1.0 to 1.0; *P* = .60).

**Figure 4.**
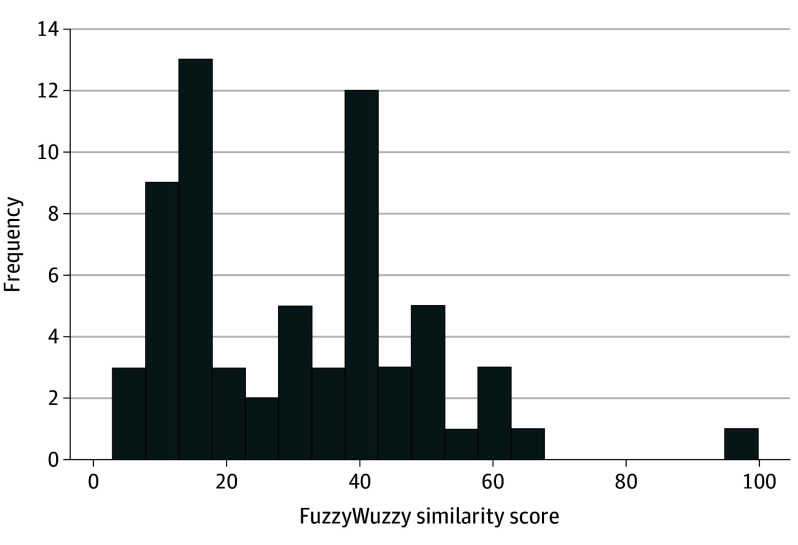
Distribution of Maximum Similarity Scores for Each Question Distribution of maximum similarity scores for each question, comparing machine-generated and human-written questions across the entire American Academy of Ophthalmology question bank. Only the highest similarity score per question was considered in the analysis.

The distribution of similarity scores is presented in Figure 4. When comparing LLM-generated questions to all questions within the same topic from the AAO question bank, the overall mean similarity score was 19.87. However, when only the highest similarity score per LLM-generated question was considered for the analysis, the overall mean similarity score increased to 29.67. Only 1 duplicate question was identified (“What is the most common inheritance pattern in inherited cases of primary congenital glaucoma?”) Notably, 95.31% of LLM-generated questions had similarity scores less than 60, suggesting very limited or no resemblance to existing items. eTable 3 in Supplement 1 shows comparison of randomly selected examples of LLM-generated questions with their most similar counterparts from the AAO question bank. The first column displays the similarity score for each matched pair.

The human-written questions had a mean (SD) readability score of 42.60 (22.84), while the LLM-generated questions had a mean (SD) score of 37.14 (22.54; *P* > .99).

Interrater reliability, assessed using ICC, indicated moderate agreement among raters (ICC, 0.63; 95% CI, 0.58-0.67; *P* < .001). To assess the consistency of expert evaluations across 2 question sources, we computed the Krippendorff α for each of the 5 evaluation domains separately for human-written and LLM-generated questions. Overall, interrater agreement was close to zero across both sources, indicating neither strong agreement nor strong disagreement, with α values ranging from −0.011 to 0.122 for human-written questions and from −0.009 to 0.126 for LLM-generated questions. Among human-written questions, the highest agreement was observed for suitability for trainees (α, 0.122), followed by discriminative power (α, 0.068) and relevance (α, 0.059), while negative agreement values were noted for clarity and specificity and appropriateness. A similar pattern was observed for LLM-generated content, where discriminative power (α, 0.126) and relevance (α, 0.094) showed relatively higher agreement.

eFigure 1 in Supplement 1 presents a comparison of the quality of human experts’ questions and those produced by the LLM. Box plots illustrate the distribution of scores for each generation method, providing insights into the variability and central tendency of the ratings. eFigure 2 in Supplement 1 shows the combined weighted mean scores for various subspecialties.

eTables 1 and 2 in Supplement 1 show examples of LLM-generated questions in the retina and optics subspecialty, alongside the AAO human-written example questions provided in the prompt. eTable 3 in Supplement 1 presents comparison of randomly selected examples of LLM-generated questions with their most similar counterparts from the AAO question bank, providing insights into the similarity scores.

## Discussion

In this study, we aimed to leverage an LLM to generate new ophthalmology board–style MCQs content and found that GPT-4 generated high-quality ophthalmology board–style MCQs, with median scores comparable to human-written questions that have undergone multiple rounds of review and revision by several experts. These findings suggest that GPT-4 holds promise as a valuable tool for enhancing examination preparation materials for ophthalmology residents and expanding question banks for ophthalmology board examinations, adding important information to the existing literature regarding AI in ophthalmology, which has focused on its potential role in clinical diagnostic aids, detection and monitoring of ophthalmic diseases, and disease prognostication and prediction. Some examples of such studies include screening for diabetic retinopathy via fundus photos, analyzing optical coherence tomography data for early detection or progression of the disease, assessing severity and etiology of corneal ulcers, screening for refractive surgery candidates,^2,3,4,5,6^ predicting glaucoma conversion,^7^ answering ophthalmic MCQs,^16,17^ answering ophthalmology-related patient queries,^18^ and case management.^19^

In addition to comparable median scores, we found a moderate intraclass agreement among raters (ICC, 0.63). Mistry and colleagues^20^ reported expert review scores of 9.8 to 9.9 out of 10 for GPT-4–generated radiology MCQs, but their evaluation involved only 2 reviewers, and no interrater analysis was presented. Law and colleagues^21^ included 24 participants in evaluating ChatGPT-4o’s ability to generate high-quality MCQs for emergency medicine examination. They also reported a moderate interrater agreement between LLM- and human-written scores (ICC, 0.62). Additionally, we observed consistently low α values when evaluating the interrater reliability for each question set separately. Our results suggest a lack of consensus and variability in expert judgments, potentially reflecting inconsistencies in rater interpretation due to the subjective nature of the evaluation criteria or differences in grader interpretation. These findings highlight the need for more structured rubrics, a larger sample size, or calibration among raters to enhance reliability in the future.

We used a similarity score to evaluate whether the LLM-generated questions were unique or novel compared to existing questions in the AAO *BCSC* Self-Assessment question bank. We found that nearly 95% of LLM-generated questions had similarity scores less than 60, indicating that most LLM questions had limited or no resemblance to existing items.

In our study, both question sets were generally appropriate for college-level readers, and human-written questions were, on average, more readable than those generated by the LLM, but this difference was not statistically significant. Although Law and colleagues^21^ did not report Flesch Reading Ease metric scores, they found that AI-generated MCQs were easier, as measured by a higher difficulty index (defined as the proportion of correct responses to each question, with a higher value indicating easier questions), suggesting LLM-written MCQs had simpler language and structure.

While our study found that GPT-4 can generate high-quality MCQs in nearly 5 seconds per question, expert review of these AI-generated questions is still essential to ensure clinical accuracy, relevance, and alignment with learning objectives. Some authors reported that reviewers typically require less than 5 minutes per question to validate and edit GPT-generated content, significantly less than the time needed to author questions from scratch.^22^ It is paramount that institutions develop standardized review protocols to streamline the validation process and maintain consistency. Additionally, access to GPT models compliant with data privacy and licensing requirements is essential, which may involve subscription costs and information technology infrastructure, and faculty will need training in prompt engineering and AI literacy to generate and refine questions effectively.

Our findings suggest that AI-enabled models can play a valuable role in augmenting the question development process within medical education. Specifically, LLMs can help fill existing gaps in question banks by generating high-quality questions for underrepresented topics, thereby expanding the breadth of available content. Additionally, these models can be used to design novel, adaptive questions tailored to individual residents’ areas of weakness, supporting more personalized and efficient examination preparation. Due to the rigorous standards of the OKAP examination, it typically takes around 3 years of full participation on the committee for an item writer to have their questions approved through the multistep review process without significant rewrites or outright rejection. By generating well-structured and grammatically sound drafts, LLMs can also reduce the time experts spend on language refinement, allowing them to focus more on evaluating clinical accuracy and pedagogical value—ultimately streamlining the content development pipeline and enhancing the quality of training programs.

### Limitations

Our study has limitations. As a single-model system, GPT-4 cannot dynamically integrate external tools or real-time data unless explicitly connected to such resources, which can constrain its adaptability in complex or evolving contexts. GPT-4’s knowledge is limited to information up to its last training cutoff and may not reflect recent developments or emerging trends. Furthermore, GPT-4’s responses may reflect biases or inaccuracies in the training data and struggle with domain-specific nuances or rare edge cases. These constraints highlight the importance of human oversight and critical evaluation when using GPT-4 for decision-making or specialized applications. Longitudinal studies examining the educational impact of adding LLM-generated content on residents’ performance testing would provide deeper insights into its efficacy and limitations. Finally, although most LLM-generated questions received low similarity scores to material already available on the AAO’s question bank, not all generated questions were unique or novel.

## Conclusions

In conclusion, an LLM generated questions that were comparable to those written by a committee of human experts through iterative review and refinement in terms of appropriateness, clarity, relevance, discriminative power, and suitability for trainees. These findings suggest that LLMs could serve as valuable and accessible resources for ophthalmology training.

## Notes

Supplement 1. eTable 1. Example of a Large Language Model (LLM)–Generated Question in the Retina Subspecialty, Alongside the AAO Human-Written Example Questions Provided in the Prompt eTable 2. Example of a Large Language Model (LLM)–Generated Question in the Optics Subspecialty, Alongside the AAO Human-Written Example Questions Provided in the Prompt eTable 3. Comparison of Randomly Selected Examples of LLM-Generated Questions With Their Most Similar Counterparts From the AAO Question Bank eResults. Results Based on “Average” Scores eFigure 1. Weighted Average Scores Assigned by 10 Expert Ophthalmologists Who Participated in the Survey to Compare Question Generation Methods Based on the Following Criteria: (a) Appropriateness, (b) Clarity and Specificity, (c) Relevance, (d) Discriminative Power, and (f) Suitability for Trainees eFigure 2. Combined Weighted Average Scores Across All Assessment Criteria for Various Subspecialties, as Assigned by 10 Expert Ophthalmologists Who Participated in the Survey Comparing Question Generation Methods

Supplement 2. Data Sharing Statement

## References

[1] Ting DSW, Cheung CYL, Lim G, . Development and validation of a deep learning system for diabetic retinopathy and related eye diseases using retinal images from multiethnic populations with diabetes. JAMA. 2017;318(22):2211-2223. doi:10.1001/jama.2017.18152 29234807 PMC5820739

[2] Kihara Y, Heeren TFC, Lee CS, . Estimating retinal sensitivity using optical coherence tomography with deep-learning algorithms in macular telangiectasia type 2. JAMA Netw Open. 2019;2(2):e188029. doi:10.1001/jamanetworkopen.2018.8029 30735236 PMC6484597

[3] Rajalakshmi R, Subashini R, Anjana RM, Mohan V. Automated diabetic retinopathy detection in smartphone-based fundus photography using artificial intelligence. Eye (Lond). 2018;32(6):1138-1144. doi:10.1038/s41433-018-0064-9 29520050 PMC5997766

[4] Yoo TK, Ryu IH, Lee G, . Adopting machine learning to automatically identify candidate patients for corneal refractive surgery. NPJ Digit Med. 2019;2(1):59. doi:10.1038/s41746-019-0135-8 31304405 PMC6586803

[5] Gholami S, Scheppke L, Kshirsagar M, ; MacTel Research Group. Self-supervised learning for improved optical coherence tomography detection of macular telangiectasia type 2. JAMA Ophthalmol. 2024;142(3):226-233. doi:10.1001/jamaophthalmol.2023.6454 38329740 PMC10853868

[6] Gholami S, Scheppke L, Kshirsagar M, ; Lowy Medical Research Institute. Enhanced macular telangiectasia type 2 detection: leveraging self-supervised learning and ensemble models. Ophthalmol Sci. 2025;5(4):100710. doi:10.1016/j.xops.2025.100710 40225407 PMC11987621

[7] Huang X, Raja H, Madadi Y, . Predicting glaucoma before onset using a large language model chatbot. Am J Ophthalmol. 2024;266:289-299. doi:10.1016/j.ajo.2024.05.022 38823673 PMC11402578

[8] Brown TB, Mann B, Ryder N, . Language models are few-shot learners. *arXiv*. Published online May 28, 2020. https://arxiv.org/abs/2005.14165

[9] Introducing ChatGPT. OpenAI. Accessed June 18, 2025. https://openai.com/index/chatgpt/

[10] Vallance C. ChatGPT: new AI chatbot has everyone talking to it. BBC. Accessed June 18, 2025. https://www.bbc.com/news/technology-63861322

[11] Kung TH, Cheatham M, Medenilla A, . Performance of ChatGPT on USMLE: potential for AI-assisted medical education using large language models. PLOS Digit Health. 2023;2(2):e0000198. doi:10.1371/journal.pdig.0000198 36812645 PMC9931230

[12] Navarro G. A guided tour to approximate string matching. ACM Comput Surv. 2001;33(1):31-88. doi:10.1145/375360.375365

[13] Flesch R. How to Write Plain English. HarperCollins; 1981.

[14] Brennan RL, Prediger DJ. Coefficient kappa: some uses, misuses, and alternatives. Educ Psychol Meas. 1981;41(3):687-699. doi:10.1177/001316448104100307

[15] Shrout PE, Fleiss JL. Intraclass correlations: uses in assessing rater reliability. Psychol Bull. 1979;86(2):420-428. doi:10.1037/0033-2909.86.2.420 18839484

[16] Mihalache A, Huang RS, Popovic MM, . Accuracy of an artificial intelligence chatbot’s interpretation of clinical ophthalmic images. JAMA Ophthalmol. 2024;142(4):321-326. doi:10.1001/jamaophthalmol.2024.0017 38421670 PMC10905373

[17] Gholami S, Wilson B, Page S, . Bridging gaps in ophthalmology education through large language models. AJO Int. 2025;2(4):100166. doi:10.1016/j.ajoint.2025.100166

[18] Tailor PD, Dalvin LA, Chen JJ, . A comparative study of responses to retina questions from either experts, expert-edited large language models, or expert-edited large language models alone. Ophthalmol Sci. 2024;4(4):100485. doi:10.1016/j.xops.2024.100485 38660460 PMC11041826

[19] Huang AS, Hirabayashi K, Barna L, Parikh D, Pasquale LR. Assessment of a large language model’s responses to questions and cases about glaucoma and retina management. JAMA Ophthalmol. 2024;142(4):371-375. doi:10.1001/jamaophthalmol.2023.6917 38386351 PMC10884943

[20] Mistry NP, Saeed H, Rafique S, Le T, Obaid H, Adams SJ. Large language models as tools to generate radiology board-style multiple-choice questions. Acad Radiol. 2024;31(9):3872-3878. doi:10.1016/j.acra.2024.06.046 39013736

[21] Law AK, So J, Lui CT, . AI versus human-generated multiple-choice questions for medical education: a cohort study in a high-stakes examination. BMC Med Educ. 2025;25(1):208. doi:10.1186/s12909-025-06796-6 39923067 PMC11806894

[22] Ch’en PY, Day W, Pekson RC, . GPT-4 generated answer rationales to multiple choice assessment questions in undergraduate medical education. BMC Med Educ. 2025;25(1):333. doi:10.1186/s12909-025-06862-z 40038669 PMC11877964

