# Supplementary material for: Leveraging Large Language Models to Generate Multiple-Choice Questions for Ophthalmology Education
Source: JAMA Ophthalmol. 2025 Oct 16;143(11):955–61. doi: 10.1001/jamaophthalmol.2025.3622 (PMC12532029; doi:10.1001/jamaophthalmol.2025.3622)
Supplement: Supplement 1. — eTable 1. Example of a Large Language Model (LLM)–Generated Question in the Retina Subspecialty, Alongside the AAO Human-Written Example Questions Provided in the Prompt eTable 2. Example of a Large Language Model (LLM)–Generated Question in the Optics Subspecialty, Alongside the AAO Human-Written Example Questions Provided in the Prompt eTable 3. Comparison of Randomly Selected Examples of LLM-Generated Questions With Their Most Similar Counterparts From the AAO Question Bank eResults. Results Based on “Average” Scores eFigure 1. Weighted Average Scores Assigned by 10 Expert Ophthalmologists Who Participated in the Survey to Compare Question Generation Methods Based on the Following Criteria: (a) Appropriateness, (b) Clarity and Specificity, (c) Relevance, (d) Discriminative Power, and (f) Suitability for Trainees eFigure 2. Combined Weighted Average Scores Across All Assessment Criteria for Various Subspecialties, as Assigned by 10 Expert Ophthalmologists Who Participated in the Survey Comparing Question Generation Methods [file jamaophthalmol-e253622-s001.pdf]

Supplemental Online Content

Gholami S, Mummert DB, Wilson B, et al. Leveraging large language models to generate multiple-choice questions for ophthalmology education. *JAMA Ophthalmol*. Published online October 16, 2025. doi:10.1001/jamaophthalmol.2025.3622

eTable 1. Example of a Large Language Model (LLM)–Generated Question in the Retina Subspecialty, Alongside the AAO Human-Written Example Questions Provided in the Prompt

eTable 2. Example of a Large Language Model (LLM)–Generated Question in the Optics Subspecialty, Alongside the AAO Human-Written Example Questions Provided in the Prompt

eTable 3. Comparison of Randomly Selected Examples of LLM-Generated Questions With Their Most Similar Counterparts From the AAO Question Bank

eResults. Results Based on “Average” Scores

eFigure 1. Weighted Average Scores Assigned by 10 Expert Ophthalmologists Who Participated in the Survey to Compare Question Generation Methods Based on the Following Criteria: (a) Appropriateness, (b) Clarity and Specificity, (c) Relevance, (d) Discriminative Power, and (f) Suitability for Trainees

eFigure 2. Combined Weighted Average Scores Across All Assessment Criteria for Various Subspecialties, as Assigned by 10 Expert Ophthalmologists Who Participated in the Survey Comparing Question Generation Methods

This supplemental material has been provided by the authors to give readers additional information about their work.

**eTable 1.** Example of a Large Language Model (LLM)–Generated Question in the Retina Subspecialty, Alongside the AAO Human-Written Example Questions Provided in the Prompt
**LLM-generated question**
Question: A 34-year-old construction worker presents to the emergency department after being struck in the eye by a nail. He has a significant subconjunctival hemorrhage, deepened anterior chamber, and severe vitreous hemorrhage. Upon examination, the intraocular pressure is normal. What is the first condition that must be ruled out in planning his management?<br><br>A. Retinal detachment<br>B. Ruptured globe<br>C. Macular hole<br>D. Angle recession
**Example AAO human-written questions used in the prompt:**
Question 1: An 18-year-old man was hit in the right eye with a bungee cord. He has a hyphema, a significant subconjunctival hemorrhage, and a vitreous hemorrhage. What condition must be ruled out first in planning management? Choose all correct choices from this list without additional explanation before or after the answer: {'1': 'retinal dialysis', '2': 'ruptured globe', '3': 'macular hole', '4': 'angle recession'}

Question 2: An eye with a penetrating injury has a corneal laceration. What would be an indication to perform an immediate vitrectomy in addition to the immediate primary wound repair? Choose all correct choices from this list without additional explanation before or after the answer: {'1': 'intravitreal foreign body', '2': 'posterior extension of laceration', '3': 'vitreous hemorrhage', '4': 'uveal prolapse'}

Question 3: A patient undergoes repair of an open-globe injury that involves a large laceration extending posteriorly. The ophthalmologist who is closing the wound has trouble visualizing the most posterior aspect, and suturing becomes very difficult. What is the next step in management? Choose all correct choices from this list without additional explanation before or after the answer: {'1': 'Continue dissecting away conjunctiva and Tenon capsule until the most posterior aspect of the wound can be closed.', '2': 'Perform immediate vitrectomy to reduce vitreoretinal traction then complete suturing of the wound.', '3': 'Leave the most posterior aspect of the wound to heal without sutures.', '4': 'Place fibrin sealant on the most posterior aspect of the wound to facilitate full closure.'}

**eTable 2.** Example of a Large Language Model (LLM)–Generated Question in the Optics Subspecialty, Alongside the AAO Human-Written Example Questions Provided in the Prompt
**LLM-generated question**
Question: A patient presents with a refractive error of -3.00 -0.75 x 120. What type of astigmatism does this patient have?<br><br>A. Compound myopic astigmatism<br>B. Simple myopic astigmatism<br>C. Mixed astigmatism<br>D. Simple hyperopic astigmatism
**Example AAO human-written questions used in the prompt:**
Example AAO questions:<br><br>Question 1: The refraction of + 2.00 + 1.50 x 90 indicates what type of astigmatism? Choose all correct choices from this list without additional explanation before or after the answer: {'1': 'against-the-rule astigmatism', '2': 'mixed astigmatism', '3': 'simple hyperopic astigmatism', '4': 'compound hyperopic astigmatism'}

Question 2: Against-the-rule astigmatism is defined as being corrected with a plus cylinder with axis between what degrees? Choose all correct choices from this list without additional explanation before or after the answer: {'1': 'between 60-120 degrees', '2': 'between 0-10 and 170-180 degrees', '3': 'between 0-30 and 150-180 degrees', '4': 'between 80-100 degrees'}

Question 3: What type of astigmatism does a patient with a refraction of + 1.00 + 2.00 x 75 degrees have? Choose all correct choices from this list without additional explanation before or after the answer: {'1': 'mixed astigmatism', '2': 'oblique astigmatism', '3': 'against-the-rule astigmatism', '4': 'with-the-rule astigmatism'}

Question 4: What type of astigmatism best describes the eye with this prescription: + 0.50 - 1.00 x 90? Choose all correct choices from this list without additional explanation before or after the answer: {'1': 'compound hyperopic', '2': 'compound myopic', '3': 'emmetropic', '4': 'mixed'}

Question 5: What term describes the refractive error in a patient with a refraction of - 3.50 + 1.50 x 180? Choose all correct choices from this list without additional explanation before or after the answer: {'1': 'simple myopic astigmatism', '2': 'compound myopic astigmatism', '3': 'simple hyperopic astigmatism', '4': 'mixed astigmatism'}

**eTable 3.** Comparison of Randomly Selected Examples of LLM-Generated Questions With Their Most Similar Counterparts From the AAO Question Bank<br>The first column displays the similarity score for each matched pair.	
**Similarity Score**	**Question Comparisons**
100	**LLM-generated Question Example:**<br><br>What is the most common inheritance pattern in inherited cases of primary congenital glaucoma?<br><br>**AAO Question Bank Match:**<br><br>What is the most common inheritance pattern in inherited cases of primary congenital glaucoma?
64	**LLM-generated Question Example:**<br><br>What is the most common inheritance pattern in inherited cases of primary congenital glaucoma?

	**AAO Question Bank Match:**<br><br>What are the gender and laterality demographic characteristics of primary congenital glaucoma?
53	**LLM-generated Question Example:**<br><br>Which electrophysiologic test is primarily used to assess macular retinal ganglion cell function?<br><br>**AAO Question Bank Match:**<br><br>What electrophysiologic test best evaluates the retinal pigment epithelium (RPE)?
47	**LLM-generated Question Example:**<br><br>Which factor is most likely to increase the risk of overcorrection during a laser ablation procedure?<br><br>**AAO Question Bank Match:**

	What organism is the most common pathogen in cases of infectious keratitis after laser in situ keratomileusis (LASIK)?

The first column displays the similarity score for each matched pair.

eResults. Results Based on “Average” Scores

Questions generated by GPT-4, a general-purpose language model, received weighted average scores of 8.40 (appropriateness), 8.44 (clarity and specificity), 8.60 (relevance), 7.67 (discriminative power), and 7.85 (suitability for trainees). In comparison, questions authored by a committee of human experts scored 8.61, 8.56, 8.66, 8.04, and 8.06, respectively (P > .05 for all individual criteria). The combined weighted average score was 8.39 for the human expert committee and 8.19 for GPT-4 (difference = 0.2; 95% CI: [-0.60, 0.2]; P = 0.52). Figure X1 presents a comparison of the quality of human experts' questions and those produced by the LLM. Boxplots illustrate the distribution of scores for each generation method, providing insights into the variability and central tendency of the ratings. Figure X2 shows the combined weighted average scores for various subspecialties. Uveitis and retina MCQs generated by the LLM received higher scores while pathology/oncology, cataract and cornea MCQs generated by the LLM received lower scores from expert ophthalmologists compared with those authored by the human experts.

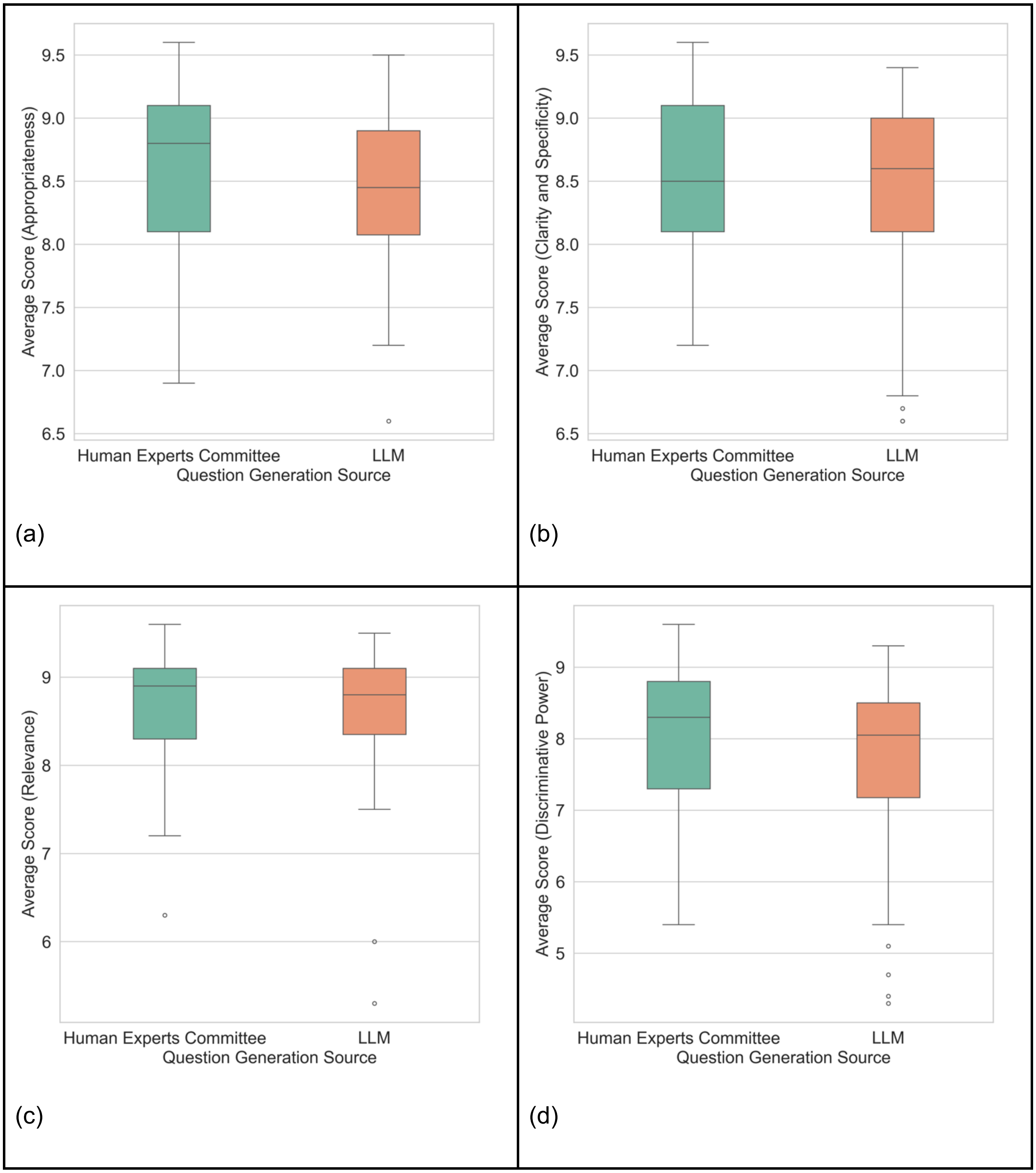

9.5
9.0
8.5
8.0
7.5
7.0
6.5
Average Score (Appropriateness)
Human Experts Committee
LLM
Question Generation Source
(a)
Average Score (Clarity and Specificity)
(b)
9
8
7
6
Average Score (Relevance)
(c)
5
Average Score (Discriminative Power)
(d)

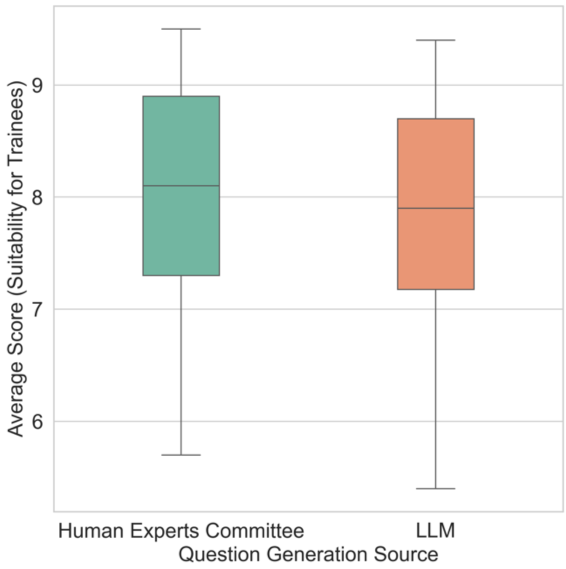

(e)

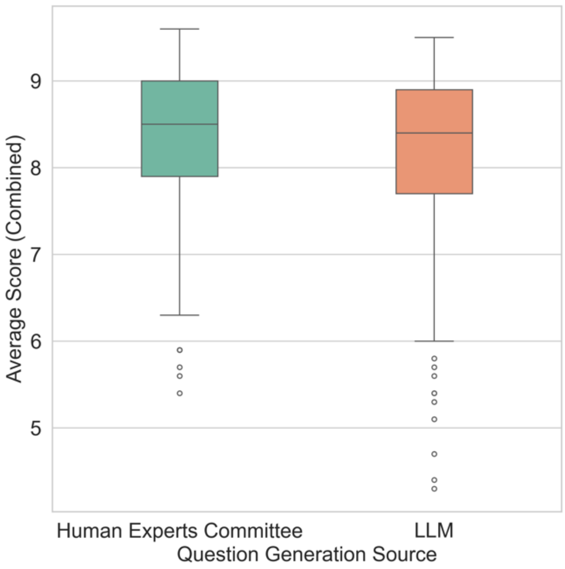

(f)

eFigure 1. Weighted Average Scores Assigned by 10 Expert Ophthalmologists Who Participated in the Survey to Compare Question Generation Methods Based on the Following Criteria: (a) Appropriateness, (b) Clarity and Specificity, (c) Relevance, (d) Discriminative Power, and (f) Suitability for Trainees

The average score across all assessment criteria is shown in (g).

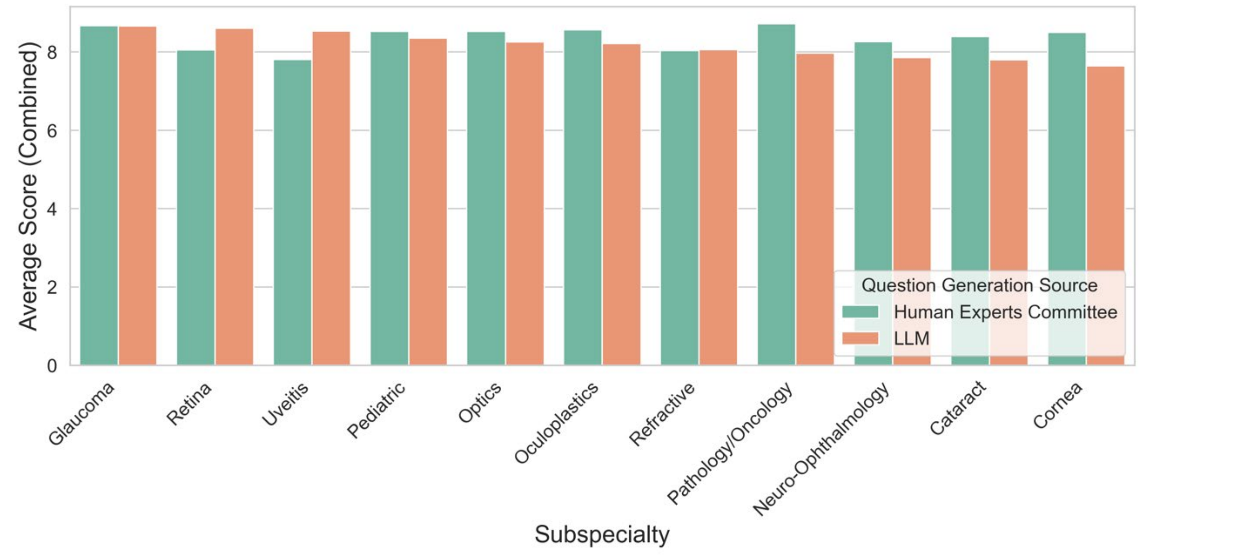

eFigure 2. Combined Weighted Average Scores Across All Assessment Criteria for Various Subspecialties, as Assigned by 10 Expert Ophthalmologists Who Participated in the Survey Comparing Question Generation Methods
