# Supplementary material for: Leveraging Large Language Models to Generate Multiple-Choice Questions for Ophthalmology Education
Source: JAMA Ophthalmol. 2025 Oct 16;143(11):955–61. doi: 10.1001/jamaophthalmol.2025.3622 (PMC12532029; doi:10.1001/jamaophthalmol.2025.3622)
Supplement: Supplement 2. — Data Sharing Statement [file jamaophthalmol-e253622-s002.pdf]

Data Sharing Statement

Gholami. Leveraging Large Language Models to Generate Multiple-Choice Questions for Ophthalmology Education. *JAMA Ophthalmol*. Published October 16, 2025. doi:10.1001/jamaophthalmol.2025.3622

Data
Data available: No

Additional Information
Explanation for why data not available: Data is protected by copyrights
